# A comparison of trends in research into home care services in Japan and Korea

**DOI:** 10.1186/1472-6955-12-18

**Published:** 2013-07-22

**Authors:** Kumi Arita, Hosihn Ryu

**Affiliations:** 1 School of Nursing, Faculty of Medicine, Fukuoka University, 7-45-1, Nanakuma, Jounan-ku, Fukuoka, Japan; 2 College of Nursing, Korea University, Anamro 145, Sungbuk-ku, Seoul 136-713, Korea

**Keywords:** Comparative study, Home care services, Japan, Republic of Korea, Research trends

## Abstract

**Background:**

The purpose of this study is to compare of the research trends for home care services in Japan and the Republic of Korea (Korea). In particular, it was compared as the research design, the method of data collection, and key words by literature review.

**Methods:**

Original articles on home care services were selected from Japanese and Korean journals published from the year of 2004 to 2008. The articles were classified, and compared in terms of the number of articles per year.

**Results:**

The research design was quite different. Quantitative research design was dominantly conducted in Korea, qualitative research design was used same level of design in Japan. In particular, outcome study was shown in Korean.

**Conclusions:**

It is suggested that future collaboration be undertaken to improve the variety of research design and method especially in both countries under the aged society. In addition, it provides information concerning research concepts, which can be applied to optimize future home care services.

## Background

Japanese and Korean community health nursing scholars gathered for the first time at the Korea-Japan Joint Conference on Community Health Nursing in November, 2007. The theme of this conference was a vision of community health nursing for aging era. A consensus was reached that continuous scientific exchange is essential in developing community health nursing in both countries, including the subspecialty of home care services [1]. In 2010, the elderly ratio in Korea was 10.2% compare in Japan was 23.1%, this is expected to rise to 14.3% in Korea and 28.4% in Japan by 2018. By contrast, the 2010 total fertility rate in Korea was 1.13 and in Japan was 1.39, which is the lowest value in the world at the time of writing [2,3]. With a rapidly increasing elderly population aged 65 years and over and a rapidly declining birth rate, the rapid increase of health expenditures has become a challenge to the government's health policy in both countries [4]. Japan introduced a system of home health care around 20 years earlier than Korea. To improve the efficacy and quality of home care services in Japan and Korea, the different practices in each country should be evaluated. Because we assumed that research findings determine clinical practice, and thus differing approaches to research may result in differing clinical practices.

In addition, the two countries have developed differing systems of nursing education. Although nursing education system extends over 4 years in both Japan and Korea, Korea was the first to introduce master and doctoral courses for nurses. In 2005, Korea also introduced a system of sending educational researchers to the United States for training in order to introduce pioneering approaches within the Korean nursing education system [5]. Both factors may have contributed to differences in the approach to research between the two countries, such as the diversity of research design. On the topic of research, In view of the cultural differences between the two countries, Japan and Korea would be expected to have differing perspectives on the appropriate approach to research into home care services.

However, no previous study has compared research trends in research into home health care services between the two countries. The aim of the present study was to review the literature on home care services in Japan and Korea in order to compare research trends and obtain baseline data concerning the nature and the extent of scientific activity.

## Methods

All academic journals relating to home care services and all publications relating to community health nursing studies published the year of 2004 to 2008 in Japan and Korea were considered for inclusion in the present literature review. Firstly, eight academic journals relating to integrated community health nursing were chosen from the 37 academic journals of the Japan Association of Nursing Academies [6]. Of these, the journals relating to home care services were chosen for consideration in the present study: There are 4 main journals relating to home care services were selected for consideration in the present study in Japan, such as, Journal of Japan Academy of Nursing Science (JJANS) [7]; Journal of Japan Society of Nursing Research (JJSNR) [8]; Journal of Japan Academy of Community Health Nursing (JJACHN) [9]; and Journal of Japan Academy of Home Care (JJAHC) [10]. Secondly, six academic journals relating to integrated community health nursing were chosen from the 22 academic journals of the Korean Society of Nursing Science. Of these, the following 3 main journals relating to home care services were selected for consideration in the present study in Korea: Journal of Korean Society of Nursing Science (JKSNS) [11]; Journal of Korean Academy of Community Health Nursing (JKACH) [12]; and Journal of Korean Home Health Nursing (JKHHN) [13]. In Japan, The number of issues per year for each of these journals varies: JJANS (4 issues); JJSNR (5 issues); JJACHN (2 issues); JJAHC (2 issues) in Japan, and JKSNS (6 issues), JKACHN (4 issues), and JKHHN (2 issues) in Korea. A literature search in Japanese and Korean journals was performed using internet homepage from each journal. The academic journal name was inputted as the search item, and the original article was inputted as the preferred paper source. A total of 118 original articles were selected from the four academic journals from Japan. All 87 original articles identified from the three Korean academic journals were selected, since all were original publications. The 205 selected articles were categorized according to publication year, research design, data collection method, sampling, sample size, and keywords. Research design and the data collection method were classified according to the Practice of Nursing Research [14]. Japanese and Korean keywords were based on the Medical Subject Headings (MeSH) thesaurus of the U.S. National Library of Medicine (NLM). However, MeSH terms as the keywords in both countries do not show exactly by authors originally. Therefore, we applied the most appropriate alternative to compare, which was to analyze the keywords presented by the authors. Original articles require keywords that have been selected from MeSH. However, a large proportion of studies use inappropriate keywords. Therefore, the present study considered the keywords used by the authors.

## Results

### Research design

The number of articles in both countries was conducted over the five year period. As would be expected, the majority of original articles were published in journals representative of home care services, such as JJAHC (44.9% of all original articles in Japan) and JKHHN (54.0% of all original articles in Korea) (Table 1). In Japan, 69.5% of articles described quantitative research, 28.7% described qualitative research, and 1.7% described outcome research. In Korea, 80.5% of articles described quantitative research, 12.5% described qualitative research, and 6.9% described outcome research. In detail, a large number of quantitative researches in Japan, content analysis of research method from qualitative method were 18.6%, which was the second highest research after the predictive level of study design with under 30 study samples from personal in-depth interview. Accordingly, Japan had the highest level of predictive study design at 31.4%, whereas Korea had the highest level of descriptive study design at 32.2% (Table 2).

**Table 1:** Comparison of the number of original articles on home care services in the main Japanese and Korean journals of the year from 2004 to 2008

**Source year**	**Japan**					**Korea**			
	**JJANS**^**1)**^	**JJSNS**^**2)**^	**JJACHS**^**3)**^	**JJAHC**^**4)**^	**Total**	**JKSNS**^**5)**^	**JKACHN**^**6)**^	**JKAHHN**^**7)**^	**Total**
2004	2	3	7	10	22	9	7	6	22
2005	4	6	5	16	31	2	7	12	21
2006	2	5	3	5	15	4	0	6	10
2007	10	2	4	15	31	0	4	11	15
2008	5	4	3	7	19	1	6	12	19
Total	23	20	22	53	118	16	24	47	87
(%)	(19.5)	(16.9)	(18.6)	(44.9)	(100.0)	(18.4)	(27.6)	(54.0)	(100.0)

1) Journal of Japan Academy of Nursing Science, 2) Journal of Japan Society of Nursing Research, 3) Journal of Japan Academy of Community Health Nursing, 4) Journal of Japan Academy of Home Care, 5) Journal of Korean Society of Nursing Science, 6) Journal of Korean Academy of Community Health Nursing, 7) Journal of Korean Academy of Home Health Nursing.

**Table 2:** Comparison of the research design

**Study design**	**Japan**	**Korea**
	**N (%)**	**N (%)**
Quantitative research		
Descriptive study	15(12.7)	28(32.2)
Instrument development (including meta analysis)	9(7.6)	3(3.5)
Secondary analysis (including literature review)	5(4.3)	3(3.5)
Program development	2(1.7)	6(6.9)
Correlative study	12(10.2)	15(17.2)
Predictive study	37(31.4)	7(8.0)
Quasi-experimental study	2(1.7)	8(9.2)
Qualitative research		
Content analysis	22(18.6)	1(1.1)
Grounded theory study	9(7.6)	2(2.3)
Phenomenological study	0(0.0)	2(2.3)
Ethnographical study	2(1.7)	0(0.0)
Q-method	0(0.0)	1(1.1)
Others (including expert opinions)	1(0.8)	5(5.7)
Outcome Research	2(1.7)	6(6.9)
Total	118(100.0)	87(100.0)

### Research methods

In Japan, 28.8% of studies involved personal in depth interviews, compared to only 6.9% in Korea. Korean studies had the highest rate of questionnaire use at 32.2%. As for nursing intervention as a prescription research, Korea had 9.2% of articles, and Japan had three articles only 2.5% (Table 3). Japan had the highest rate of convenience sampling at 83.9%, compared to 67.8% in Korea. Total sampling was reported in 15.0% of articles in Korea, and 2.5% of articles in Japan (Table 4). Sample sizes range from 100 to 499 was reported in 38.1% of articles in Japan, and 37.9% of articles in Korea. In Korea, 12.6% of articles reported sample sizes exceeding 1,000. Samples sizes of 30 or less were reported in 33.9% of articles in Japan, and 8.0% of articles in Korea (Table 4).

**Table 3:** Comparison of the research method used for data collection

**Method of data collection**	**Japan**	**Korea**
	**N (%)**	**N (%)**
Questionnaire survey	29(24.6)	28(32.2)
Mail survey	31(26.3)	15(17.2)
Personal in-depth interview	34(28.8)	6(6.9)
Focus group interview	1(0.8)	3(3.4)
Nursing intervention	3(2.5)	8(9.2)
Program development	2(1.7)	3(3.4)
Analysis of nursing records	5(4.3)	12(13.8)
Secondary analysis	11(9.3)	3(3.4)
Expert opinions	1(0.8)	8(9.2)
Others	1(0.8)	1(1.2)
Total	118(100.0)	87(100.0)

**Table 4:** Comparison of sampling method and sample size

**Sampling method & Sample size**	**Japan**	**Korea**
	**N (%)**	**N (%)**
Sampling method		
Total sampling		3(2.5)	13(15.0)
Randomized sampling	11(9.3)	7(8.0)	
Convenience sampling	99(83.9)	59(67.8)	
Literature review	5(4.3)	1(1.2)	
	None	0(0.0)	7(8.0)
Total	118(100.0)	87(100.0)	
Sample size			
Under 30	40(33.9)	7(8.0)	
30-99	21(17.8)	22(25.3)	
100-499	45(38.1)	33(37.9)	
500-999	3(2.5)	6(6.9)	
1000 and above	4(3.4)	11(12.6)	
None	0(0.0)	7(8.0)	
Literature	5(4.3)	1(1.2)	
Total	118(100.0)	87(100.0)	

### Keywords

As a result of selecting a keyword from original articles which had 261 keywords in Japan and 159 keywords in Korea (Table 5). We analyzed all key words presented by the authors, and then classified them into all the following seven research main concepts: system and home care services, study participants, symptom and disease, health status and health behavior, intervention, management, outcome or evaluation, and family and social support. In both countries, most keywords were related to the concept of system and home care services. The rate of intervention, management, and outcome or evaluation research was higher in Korea compared to Japan. The articles from Japan used diverse keywords, such as mentality, cancer, remote medical treatment, day care, and night care. The Korean articles included keywords relating to nurses and housebound clients, and few were related to family. The Korean articles also contained a keyword concerning advanced practice nursing. Symptom and disease are keywords that relate to health problems or conditions. In the Japanese articles, diverse subjects were discussed, including dementia, cerebrovascular disorders, and stress. The main topics in the Korean articles were stress and mental disorder, anxiety, severe depression, and topics related to chronic disease. Health status or health behaviour is keywords used to express attitude to target health. In Japan, many keywords reflected the moral aspects of mental health, grief, or health behaviour. The articles from Korea contained many keywords reflecting recognition of the importance of cognition and self efficacy. Articles describing outcome or evaluation research used keywords that showed how the subject had chosen to react to a specific problem or intervention in Korea. In terms of the family and social support concept, the respective articles from Japan concerned family nursing, family relationships, and social support, while those from Korea concerned social support and family burden.

**Table 5:** Comparison of the keywords in original articles concerning home care services

**Category**	**Japan**		**Korea**	
	**N (%)**	**Keyword (n)**	**N (%)**	**Keyword (n)**
System/Management/Services	119(45.7)	Home care services(78), Terminal care(9), Patient-centered care(7), Mental health care(5), Home nursing agencies(3), Long term care insurance(3), Wilderness medicine(2), Medical care(2), Universal precaution(2), Community networks, Day care, Health resources, Night care, Critical pathways, Nursing care management, Patient care team relationship, Staff development	62(39.0)	Home care services(32), Nursing homes(2), Long term care insurance(2), Ethics(2), Job description(2), Nursing activity(2), Total quality management(2), Role(2), Accreditation, Hospice, Hot lines, Telemedicine, Curriculum, Equipment safety, Moral, International classification for nursing practice(ICNP), Organizations, Role conflict, Policy, Risk factor, Task, Time management, Ubiquitous, U-healthcare
Subjects	43(16.5)	Caregivers(15), Disabled persons(7), Care manager(4), Home nursing patients(4), Family(3), Home helper(3), Aged, Congresses as topic, Inpatients, Peer groups, Occupational groups, Self-help groups, Spouses	25(15.7)	Community health nurse(9), Aged(6), Advanced practice nurse(2), Caregivers(2), Home nursing patients(2), Emigrants and immigrants, Living alone, Students, Women
Symptom/Disease	48(18.4)	Dementia(5), Stress(5), Cerebrovascular disorders(4), Bereavement(3), Fatigue(2), Femoral neck fractures(2), Pressure ulcer(2), Terminally ill(2), Schizophrenia(2), Aphasia(2), Accidental falls, Amyotrophic lateral sclerosis, Cerebral infarction, Disease progression, Hemiplegia, Hip joint, Hypoesthesia, Pain, Muscular dystrophy, Chronic obstructive pulmonary diseases, Renal insufficiency, Rheumatoid, Parenteral nutrition, Renal dialysis, Surgical stomas, Sleep arousal disorder, Unilateral spatial neglect, Urinary incontinence, Ventilators	18(11.3)	Stress disorders(4), Accidental falls(2), Anxiety(2), Depression(2), Stroke(2), Diabetics, Heart failure, Myocardial infarction, Neoplasm, Renal dialysis, Pain
Health Status/Behaviour	21(8.0)	Mental health(5), Grief(3), Acclimatization(2), Affect, Attitude, Behaviour, Empowerment, Food, Human activities , Life style changes, Self-assessment, Sleep, Self concept, Walking	18(11.3)	Cognition(3), Perception(2), Self efficacy(3), Health Promotion(3), Attitude, Fertility, Exercise, Health status, Power, Sexual behaviour, Sleep
Intervention	9(3.4)	Rehabilitation(3), Self support program(2), Exercise therapy, Oxygen inhalation therapy, Hygiene, Infection control	10(6.3)	Education(4), Rehabilitation(2), Drug therapy, Foot reflex, Exercise therapy, Massage
Outcome/Evaluation	15(5.7)	Patient satisfaction(4), Geriatric assessment(2), Life style(2), Sleep(2), Job satisfaction, Daily living activities, Evaluation research, Quality of life, Risk assessment	22(13.8 )	Satisfaction(7), Self care(3), Quality of life(3), Outcome assessment(3), Daily living activities(2), Nutrition assessment(2), Evaluation, Quality management index
Family/Social support	6(2.3)	Family nursing(2), Family relations(2), Child rearing, Social support	4(2.5)	Social support(2), Family burden, Family function
Total	261(100.0)		159(100.0)	

Note: (n) = the number of keywords.

## Discussion

A difference in the number of original articles on home care services between the two countries was expected. One possible explanation for the lower number of original articles in Japan is their differing history of nursing education. The nursing education system of both countries was established in 1952. In Japan, a master's course was established in 1979, and the doctoral course was established in 1988. In Korea, a master's course was established in 1960 and a doctoral course was established in 1978 [5]. The establishment of university nursing facilities in Japan increased following the introduction of the 1992 Act on Assurance of Work Forces of Nurses and Other Medical Experts, which stipulated the nature of the nursing profession and the necessity for recruiting talented individuals [15]. In Korea, the home health care system differs slightly from the American home health care system or the Japanese visiting care system [16] in that it consists of three related service programs, with each service having its own unique law and regulations. The first service program is called the home care nursing (HCN) program, which serves patients who have been discharged early from hospital; this program was established by the Medical Care Act in 2000. The second service program is called the visiting care nursing (VCN) program and it was developed for managing homebound elderly patients with chronic diseases; it was established by the Long-term Care Security for the Elderly Act in 2007. The third program is called the home health care (HHC) program and it was developed to prevent illness and promote health in vulnerable people in the community; it was established in 1990 by the Community Health Act. Furthermore, the Korean home care services journal (JKHHN) was established in 1995. Although few studies of home care services have been reported to date in Korea, the Korean research effort commenced rapidly in response to a low birth rate and increased longevity, and we thus predict that the investigation of home care services will now increase.

Japanese research into home care services mainly involved content analysis, small sample sizes, and convenience sampling. Furthermore, a large proportion of studies involved personal in-depth interviewing. The key word of home care service research in Japan was “symptom and condition” dominate. Thus, the research tended to focus on an individual or a small group, the needs of the subject, and a description of the phenomenon. The secondary aim of the research seemed to address why a specific event happened in terms of its relationship with this phenomenon. This is with the contents of the care, or service, and the study for predicting the concrete result in a fixed situation. This is the main research design used in home care services in Japan. Although the early stages of research into practice tend to involve analysis of the condition of study participants, Shimanouchi pointed out that intervention studies become more common as practice advances [17]. These studies may assess factors such as service organization, the content of care, cost, and outcome evaluation. According to Burns & Grove [14], correlation studies, quasi- experimental studies, and experimental studies are performed once the level of knowledge concerning a particular study problem has increased. At the time of writing, the next stage for researchers in the field in Japan is to determine the quality of home care services, since the quantitative evaluation of these services is now demanded. Evidence-based knowledge concerning useful aspects of home care services, such as the effects of home care service practice, must be compiled [18]. As mentioned by Uchida, an outcome research to evaluate the quality of home care services and caring method that will extend the concept of outcome to system establishment and a short period of economic evaluation, as well as the development of a care management method for planning the promotion of independence and preventive measures among healthy populations [19]. By contrast, Korean studies largely applied a descriptive study design, and few qualitative studies have been performed. Korean keywords included many items relating to intervention, management, and outcome. Studies of nursing intervention and outcome evaluation require a flexible approach, and should be carried out in accordance with the prevailing legal system or population dynamics.

## Conclusion

The present findings suggest that community nursing academics in Japan and Korea need to conduct original research using advanced and variety research designs and methods in order to optimize their respective home care services. In Japan, advanced research design, such as prescriptive research design or outcome research, should be introduced, based on knowledge accumulated through content analysis research. Quasi-experimental intervention studies are therefore necessary. In Korea, detailed qualitative research, such as that performed in Japan, is warranted. Combining evidence from home care service research obtained in both countries would be advantageous in terms of service development. It is suggested that future collaboration be undertaken to improve the variety of research design and method especially in both countries under the aged society. In addition, it provides information concerning research concepts, which can be applied to optimize future home care services research. These data suggest that research methodology remains at a primary level in both countries. In Japan, more research into the prescribed level or outcome of home care services is warranted. In Korea, more qualitative research is needed. These changes should lead to improved home care services in these countries.

## Competing interests

The authors declare that they have no competing interests.

## Authors’ contributions

HR planned the research design, acquired the data, coordinated the study, analyzed the data, interpreted the results, wrote and revised the manuscript supported by the Korea Research Foundation Grant funded by the Korean Government”. KA responsible for data collection, analyzed the data, interpreted the results, wrote and revised the manuscript. Both authors read and approved the final manuscript submitted for publication.

## Pre-publication history

The pre-publication history for this paper can be accessed here:

http://www.biomedcentral.com/1472-6955/12/18/prepub
